# Patient Experiences and Perceptions with Infections Due to Multidrug-Resistant Organisms: A Systematic Review

**DOI:** 10.3390/pathogens13090817

**Published:** 2024-09-22

**Authors:** Mousa M. AlRawashdeh, Angela Ishak, Ahmed Al-Bunnia, Aris P. Agouridis, Theodore Lytras, Nikolaos Spernovasilis, Constantinos Tsioutis

**Affiliations:** 1 School of Medicine, European University Cyprus, 2404 Nicosia, Cyprus; mr181673@students.euc.ac.cy (M.M.A.); aa191323@students.euc.ac.cy (A.A.-B.); a.angouridis@euc.ac.cy (A.P.A.); t.lytras@euc.ac.cy (T.L.); 2 Department of Internal Medicine, Henry Ford Health System, Detroit, MI 48202, USA; aishak1@hfhs.org; 3 Department of Internal Medicine, German Medical Institute, 4108 Limassol, Cyprus; 4 Department of Infectious Diseases, German Medical Institute, 4108 Limassol, Cyprus; nikolaos.spernovasilis@goc.com.cy

**Keywords:** anger, anxiety, CPE, education, isolation precautions, MRSA, patient communication, psychological impact, VRE

## Abstract

Infections by multidrug-resistant organisms (MDROs) pose significant public health challenges, including increased mortality rates, healthcare costs, and significant impacts on the quality of life for patients. Utilizing a systematic review methodology adhering to PRISMA guidelines, we performed a comprehensive search across three databases, identifying 20 relevant studies that investigated the psychological effects of infections due to MDROs on hospitalized adults. The primary outcomes examined included depression, anxiety, and other psychosocial impacts, while secondary outcomes included patient and caregiver understanding of the infection. Findings revealed consistent associations between contact isolation due to MDRO infections and heightened levels of depression and anxiety among patients, although evidence regarding the impact on anger was mixed. Other psychological aspects, such as feelings of stigmatization and reduced healthcare provider interactions, were also recorded. The current systematic review highlights the importance of addressing these psychological effects through holistic, patient-centered care approaches, emphasizing the need for better communication and comprehensive education for both patients and healthcare providers. Our findings suggest that mitigating the psychological burden of MDROs can enhance overall patient care and outcomes and call for further research to optimize care strategies for patients hospitalized for infections due to MDROs.

## 1. Introduction

Healthcare and public health organizations worldwide prioritize preventing and controlling the spread of infections, especially given that infections involving multidrug-resistant organisms (MDROs) are linked to increased mortality rates and increased healthcare costs [1,2]. Multidrug-resistant organisms can be defined as pathogenic strains that are resistant to at least three classes of antibiotics at the same time [3]. Multidrug resistance ranks among the top three challenges faced by public health worldwide and most often occurs due to overuse or misuse of antimicrobials, substandard practice of transmission precautions, or less commonly may arise naturally over time through various mechanisms [4,5]. Additionally, a study revealed that MDRO infection-related hospitalizations increased a staggering 359% during a 10-year period from 1997 to 2006, especially in individuals younger than 18 years old [6]. Annual deaths in Europe and the United States associated with MDRO infections are estimated to be over 30,000 in each region [7]. In addition to health outcomes, MDRO infections continue to cause a significant amount of incremental costs in public health systems [8]. Furthermore, patients with MDRO infections have a more complicated disease course and may have up to five times longer mean length of stay in inpatient hospital settings when compared to patients with infections without MDRO [9]. To address the challenge of antibiotic resistance, autovaccine therapy has been shown to be a viable alternative to decrease recurrence and reduce the use of antifungals, thereby possibly decreasing the risk of pathogens developing resistance to common treatment modalities [10]. Other modalities include using bacterial lysates such as OM-85 BV, which has been shown to considerably reduce the use of antibiotics, and COPD exacerbations, which are also likely to reduce the emergence of antibiotic resistance [11].

In addition to elevated mortality rates and diminished quality of life directly attributable to infections, individuals with MDROs face significant psychological consequences, either directly from the infection or due to effects arising from social isolation and other measures directed at reducing MDRO transmission [12,13].

As the prevalence of MDROs continues to increase with adverse short- and long-term outcomes, understanding the mental toll on patients with MDRO infections is becoming more important, thus rendering the need for exploring strategies to improve patient experiences with MDRO infection even more apparent.

Through an exploration of the socio-cultural aspects intertwined with the experiences, knowledge, and perceptions of patients affected by MDROs, the current systematic review aims to explore the psychological impacts caused by MDRO infections. By understanding and seeking out improvements in the mental health implications of MDRO infections, we can pave the way for more holistic, systemic, and patient-centric approaches in both clinical management and public health strategies among policymakers.

## 2. Materials and Methods

### 2.1. Search Strategy

A systematic review was performed in accordance with the Preferred Reporting Items for Systematic Reviews and Meta-Analyses (PRISMA) guidelines [14]. Three databases, PubMed, Elsevier Scopus, and Web of Science, were searched for relevant articles until 1 March 2024. A medical subject headings (MeSH) term and keyword search of each database was performed. The search strategy included the following terms: “MDRO” or “multi drug resistant bacteria”, “hospital acquired”, “patient experience”, “psychological aspects of illness”, “emotional impact”, “case-control study” or “observational study”. The precise search strategy was as follows: (young adult [MeSH] OR middle age OR age 80 and over OR age 65 and older) AND (MDRO OR resistant bacteria OR multi drug resistant bacteria* OR multi drug resistant pathogen OR MRSA OR VRE OR carbapenem resistant bacteria OR drug resistance, microbial [MeSH]) AND (patient experience* OR patient attitude* OR effect on patients OR patient well-being OR patient psychology OR patient mental being OR emotional effect OR emotional impact OR patient experience OR patient perception OR psychological aspects of illness) AND (case-control study OR case control study OR observational study OR case series) AND (hospital acquired OR hospitalisation OR hospitalized OR healthcare system OR inpatient [MeSH]). This is better delineated in Table 1.

### 2.2. Study Selection

Studies were included if they assessed the impact on and perceptions of hospitalized adult patients (≥18 years of age) who were diagnosed with an MDRO-related infection during hospitalization. Studies had to be written in the English or Greek language, or English translations needed to be available. There were no restrictions on whether study participants were under isolation precautions or not. All infections caused by MDRO bacteria were included, apart from infections caused by mycobacteria species. Studies that included only patients with colonization or carrier state of MDRO were also excluded.

Titles and abstracts of all retrieved articles were evaluated to determine relevance using Rayyan [15]. Two investigators (M.M.A. and A.I.) performed the literature search and the screening. Any disagreements were resolved by the study supervisor (C.T.). Studies with irrelevant titles and/or abstracts were excluded, whereas relevant studies were assessed in full text and included if they fulfilled the inclusion criteria. The reference lists of the shortlisted articles and other reviews on the topic were reviewed to identify potentially relevant articles missed by the electronic search.

### 2.3. Quality Appraisal

To appraise the quality of the included studies, the Joanna Briggs Institute (JBI) critical appraisal tools/Briggs Institute checklists were used for the qualitative studies and quantitative (cross-sectional or quasi-experimentation) studies [16], while the Newcastle–Ottawa scale was used for other quantitative studies such as case-control or cohort studies [17]. Finally, for mixed-methods studies, the Mixed Methods Appraisal Tool version 2018 was used [18]. Two authors (A.I. and M.M.A) independently appraised the studies, and any discrepancies were addressed with a third author (C.T.). The results from the quality appraisal can be found in Tables S1–S6.

### 2.4. Data Extraction

Data were extracted and placed on an Excel^®^ 16.89 spreadsheet by two researchers (M.M.A. and A.I.). The following data were extracted: (1) primary author name, (2) publication year, (3) country of origin, (4) methodology, (5) population characteristics, and (6) study findings.

### 2.5. Study Outcomes

The primary outcomes of interest were the psychological (such as depression, anxiety, and loneliness) and psychosocial impact in patients with a history of infections with MDRO. Other outcomes of interest were the participants’ or caregivers’ understanding of their infection or clinical conditions.

## 3. Results

### 3.1. Literature Search

**Figure 1:**
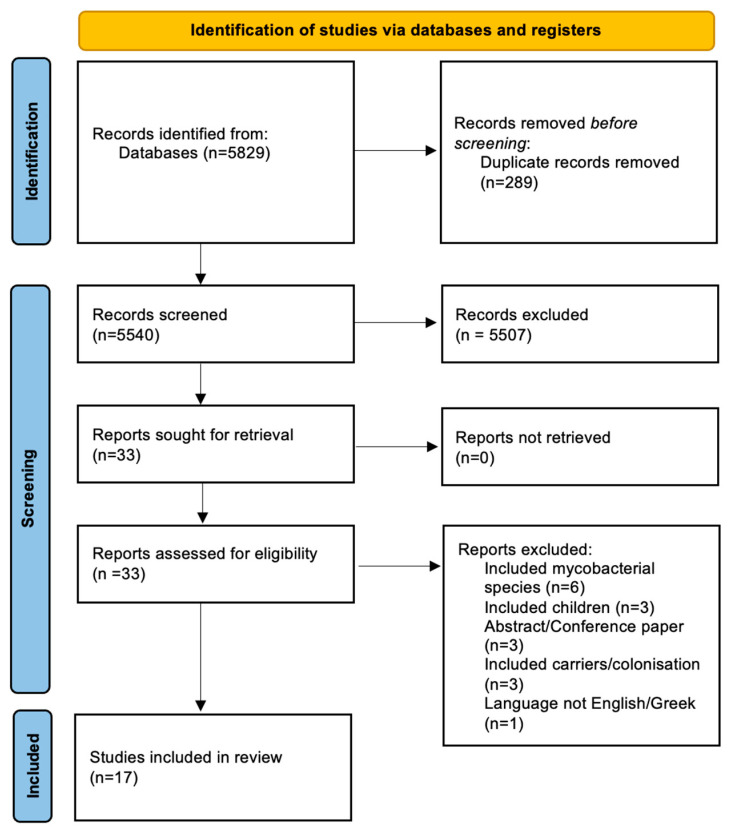
Preferred Reporting Items for Systematic Reviews and Meta-Analyses (PRISMA) flowchart reporting the search strategy results with reasons for exclusion.

The flow and selection of studies from our search strategy are summarized in Figure 1. An electronic literature search from three different databases (PubMed, Scopus, and Web of Science) retrieved 5829 articles, of which 289 were duplicates. After screening abstracts and titles, 33 were retrieved for full-text evaluation. Of the 33 manuscripts, 16 were excluded for failing to satisfy the inclusion criteria. The reasons for exclusion included studies reporting on multidrug-resistant mycobacteria and those performed in children. Thus, a total of 17 observational studies were included in the final analysis.

### 3.2. Study and Population Characteristics

Table 2 summarizes the characteristics of the included studies and the population the studies targeted. In summary, the seventeen studies included in the systematic review were conducted in various countries: six studies were in the United States, four in the United Kingdom, two in Sweden, and one each in North America (encompassing USA and Canada), Turkey, New Zealand, Australia, and Germany. Nine studies were quantitative and assessed psychological distress with various validated psychometric tests such as the Abbreviated Mental Test Score, the Barthel Index, the Profile of Mood States, the Geriatric Depression Scale, the Hospital Anxiety and Depression Scale (HADS), the Multidimensional Scale of perceived Social Support (MSPSS), the Perceived Stress Scale (PSS), Euroqol Questionnaire tests, the Hamilton Anxiety Rating Scale (HAM-A), and the Hamilton Depression Rating Scale (HAM-D). Eleven included studies were of a qualitative design, utilizing questionnaires and semi-structured and face-to-face interviews. Controls in this review consisted of patients without MDRO and patients not in isolation, including individuals diagnosed with infectious diseases not requiring contact precautions. A total of 1714 controls were included among nine studies which contained controls in their methodology [19,20,21,22,23,24,25,26,27]. One study with controls, however, did not mention the exact number of controls participating in the study [24].

### 3.3. Findings from Quantitative Studies

#### 3.3.1. Depression

Studies investigating the psychological effects of isolation brought on by MDROs have consistently shown links between patient isolation and higher levels of depression. A study performed by Tarzi et al. assessed the presence of symptoms of depression in MRSA-positive patients in contact isolation compared to those of MRSA-negative controls who were not placed in isolation [27]. Their study reported that patients in contact isolation had significantly higher depression levels than non-isolated patients (t = 3, *p* < 0.01) [27]. Similarly, isolated patients with MRSA or VRE infections demonstrated significantly higher depression scores than controls (*p* < 0.01), according to Catalano et al. [19]. These findings were also supported by Soon et al., who reported that isolated patients had noticeably higher depression levels than controls (t = 3.731, *p* < 0.01) [25]. Despite these consistent findings, Findik et al. reported no statistically significant difference in the overall depression outcomes between isolated and non-isolated patients; however, they did observe that women and those with lower incomes showed higher rates of depression within the isolated group [22].

#### 3.3.2. Anxiety

Several studies have been conducted to identify the effects of isolation on anxiety levels when MDRO infections are present. Comparing isolated MRSA-positive patients to their non-isolated counterparts, Tarzi et al. reported that those with the infection had higher anxiety levels (t = 2.98, *p* < 0.01) [27]. These findings were supported by Catalano et al., who found that after one to two weeks, isolated patients with MRSA or VRE had higher anxiety scores than controls (*p* < 0.01) [19]. These results are also supported by Soon et al., who reported that isolated patients had much higher anxiety levels than controls (t = 4.841, *p* < 0.001) [25]. However, Day et al. found no evidence of a significant correlation (OR 0.8, 95% CI 0.7–1.1) between anxiety and contact precautions in the non-ICU group [29].

#### 3.3.3. Other Psychological Aspects

Beyond anxiety and depression, various psychological dimensions have been investigated in relation to isolation resulting from MDROs. Evans et al. investigated the frequency of visits by healthcare professionals using a matched cohort observation study, reporting that compared to non-isolated patients, isolated patients were seen less frequently by medical professionals (*p* < 0.01) [21].

In order to investigate vital sign recording, Stelfox et al. used a two-matched cohort study design with 78 isolated patients compared to 156 control patients [26]. Although the difference was not statistically significant (*p* = 0.08), it was found that isolated patients were more likely to have incomplete vital sign recordings or days with no vital sign recordings at all [26].

A retrospective cohort study performed by Day et al. included 28,564 general hospital patients, 3138 of which were on contact precaution, and 7548 of them were in the intensive care unit. In the non-ICU group, they identified that contact precautions were associated with depression (odds ratio (OR) 1.4, 95% confidence interval (CI) 1.2–1.5) but not with anxiety (OR 0.8, 95% CI 0.7–1.1) [29]. Similarly, Day et al. discovered in a prospective frequency-matched cohort research that contact precautions had no serious association with feelings of concern, anger, happiness, or depression [20].

### 3.4. Findings from Qualitative Studies

#### 3.4.1. Depression and Anxiety

Multiple qualitative investigations have explored the subjective experiences and thoughts of patients who have been isolated as a result of MDROs, providing insight into the complex emotional effects that go beyond quantitative assessments.

Semi-structured interviews with 19 MRSA-infected hospitalized patients in source isolation were performed by Newton et al. Most participants had no awareness of the meaning of MRSA, although half of the participants thought it was “serious”. Patients did not exhibit considerable emotional distress that might be directly linked to isolation, even when they were unaware of its aim [32]. Similarly, ten adult patients hospitalized with MRSA and accommodated in single rooms for a minimum of three days were interviewed by Barratt et al. [28]. Patients who were isolated described feeling guilty, angry, frustrated, contaminated, and stigmatized. Additionally, they emphasized how isolation affects social interactions and staff connections, leading to a diverse emotional experience [28]. Studying six adult patients hospitalized in single rooms due to infection with MRSA, Skyman et al. found that the patients felt alone, stigmatized, ashamed, and confined. Moreover, patients illustrated psychological distress when criticizing staff members for not washing their hands properly and by expressing uncertainty and a lack of knowledge regarding their MRSA infection [33].

#### 3.4.2. Understanding and Coping

Kennedy and Hamilton’s study found that 50% of patients’ moods were negatively impacted by contact isolation after interviewing 16 MRSA-positive patients and their matched controls. However, 30% of the participants claimed that being isolated helped them manage their illness, demonstrating the variability in perceptions of contact isolation throughout participants. Nevertheless, patients who were alone reported a marked rise in anger, indicating the potential emergence of complicated emotional feelings [24]. Similarly, in an interview with 14 MRSA-colonized patients, Lindberg et al. reported that the infection caused significant psychological distress, concerns, and limitations in everyday living [31]. The differing emotional effects of isolation were reflected in the participants’ varying levels of satisfaction with healthcare contacts [31].

#### 3.4.3. Communication and Information Gaps

In one study, 14 patients with an MRSA diagnosis participated in qualitative interviews following hospital discharge [35]. Poor information provision caused uncertainty and distress in many patients, inside and outside of the hospital [35]. Watson et al.’s study assessed the level of information and understanding among staff and patients. Interestingly, 33% of participants did not know they had an MDRO, 70% of individuals did not know they were infected, and 23.3% did not know how long the infection would last [13]. Furthermore, just 35% of employees said they would know exactly what to do in the event that a patient who tested positive for CPE was admitted into their care [13].

## 4. Discussion

Several studies have sought to evaluate the anxiety and depression levels of patients diagnosed with MDROs who were placed in hospital contact isolation units as a result of their infection. One study highlighted the detrimental psychological effects of isolation measures by reporting significantly higher HAD scores among isolated patients compared to non-isolated individuals. Further support for the link between contact isolation in those patients and subsequent negative psychological outcomes was provided by a study conducted by Catalano et al., in which heightened levels of anxiety and depression were illustrated with higher HAM-A and HAM-D among isolated patients infected with MDRO compared to controls [19]. Patients reporting psychological distress raise concerns regarding potential barriers to patient care and overall health outcomes. A study by Hays et al. reported that individuals with depressive symptoms have functional and well-being limitations that are on par with or exceed those of patients with chronic illnesses, suggesting that depression causes impairments that may significantly worsen the physical and psychological harm brought on by MDROs [36]. However, one study performed by Wassenberg et al. reported no differences in anxiety and depression in colonized patients compared with controls [37]. A meta-analysis by Prina et al. revealed that hospitalized patients who experience depressive symptoms are more likely to have a prolonged hospital course and are at an increased risk for readmission [38]. Furthermore, patients with depressive symptoms have a higher chance of being admitted to the hospital, and there is a dose-response relationship between the severity and frequency of infections and depressive symptoms [39]. To improve patient outcomes and optimize the delivery of care, healthcare providers must take proactive steps with tailored support systems for patients in contact isolation to optimize care delivery and patient outcomes. Adhering to reasonable quarantine durations while avoiding unnecessary extensions may mitigate psychological consequences [40]. Additionally, offering support groups to patients in contact isolation can provide emotional support, validation, and empowerment, while reducing feelings of loneliness [41].

Though anxiety and depression were frequently noted as themes among individuals in contact isolation, the association between anger and patients with an MDRO infection varied across several studies. According to a study by Tarzi et al., which found no statistically significant rise in anger levels between isolated and non-isolated groups, anger may be less common in isolated groups than anxiety and despair [27]. Kennedy and Hamilton’s study, however, revealed a statistically significant increase in anger among isolated individuals [24]. These findings highlight the need for recognizing and treating the negative psychological consequences in patients undergoing contact isolation with MDRO infection because of the differences in the patients’ emotional responses to isolation measures. Prioritizing a comprehensive approach is imperative for healthcare practitioners to tackle these potentially detrimental psychological shifts.

Two studies compared the interactions between healthcare providers and patients in contact isolation and patients who were not isolated and without an MDRO infection. Evans et al.’s study revealed that isolated patients had fewer recorded visits from healthcare providers compared to non-isolated patients, which may lead to worsened psychological effects and foster feelings of neglect and stigma [21]. Additionally, one study reported that patients in contact isolation were more likely to have incomplete vital sign recordings or days without any vital sign recordings at all [26]. These findings emphasize the importance of providing patients in contact isolation with fair and equitable access to medical care to reduce any possible harm to their well-being. Concerns are also expressed regarding the differences in clinical surveillance and its applicability to patients under contact isolation due to an MDRO infection. These findings demonstrate the importance for healthcare professionals to ensure that patients undergoing contact isolation have fair and equitable access to care to lessen the negative toll of MDRO infections. Disparities in the way medical treatment is delivered could increase patients’ feelings of isolation, neglect, anxiety, and stigma. In addition, disparities in healthcare delivery may worsen feelings of loneliness, neglect, anxiety, and stigma.

Several studies highlighted the lack of patient knowledge and perceptions regarding their MDRO infection, revealing the apparent lack of understanding of their infection in several patients led to feelings of fear, confusion, and exacerbated anxiety [32,34]. Furthermore, Watson et al. reported that the lack of comprehensive knowledge about MDRO infections and subsequent contact isolation was also observed among healthcare providers, which ultimately led to inconsistencies in adequately and safely following contact isolation protocols and further gaps in knowledge about the infection among patients due to the lack of education from healthcare providers [13]. Moreover, providing education and training about infections and infection control has been reported to facilitate management and lead to improved patient adherence to treatment and an improvement in psychological distress [42,43]. The findings indicate the importance and need for providing comprehensive education to both healthcare providers and patients to optimize the delivery of care and alleviate the consequences of anxiety and confusion. Tailoring patient education by addressing their concerns and perceptions, providing easily understandable information about MDRO transmission, infection, and management, especially regarding contact isolation, may diminish feelings of confusion and anxiety about their diagnosis and management. Moreover, improving patient knowledge may lead to enhanced compliance with contact isolation and further management of their care. Healthcare institutions may initiate training initiatives for healthcare providers to increase knowledge about MDRO infections, specifically tailored to infectious disease control and patient care.

Another common theme reported by numerous MDRO patients in contact isolation in several studies is the feeling of stigma, perceived contamination or “feeling dirty”, and the impact on social relationships and activities. The studies by Barratt et al., Skyman et al., and Baron et al. revealed the significant adverse consequences of stigma and social isolation were apparent, leading to further psychological distress by heightening levels of anger, loneliness, and guilt [12,28,33]. These feelings may impede the patient–doctor relationship and exacerbate the course of disease for the patient. Fostering a supportive environment and community with an emphasis on alleviating stigmatization could be a potential element of improved patient care and outcomes.

Isolation measures can significantly disrupt patients’ social relationships and activities, further contributing to their psychological distress. Barratt noted strained relationships between isolated patients and healthcare staff due to restricted social interactions and perceived stigma. Goldsack et al., reported concerns about limited visitation and reduced social interactions during isolation, highlighting the need for strategies to maintain patients’ social connections and support networks while in isolation [30].

One study reported that isolation from social relationships and activities led to frustration and strained relationships with healthcare providers [28]. Similarly, Goldsack et al. raised concerns about social isolation, highlighting that patients in social isolation felt “having certain rights and privileges limited” [30]. Furthermore, Chen et al. reported that patients with supportive family members who supervised medication and offered spiritual encouragement led to improved adherence to medication and improved health outcomes, further highlighting the importance of maintaining patient–family relationships [43]. Healthcare providers must recognize and address the significant effects of contact isolation on patients’ personal and social relationships. Implementing a holistic approach that compounds and prioritizes mental health support and family-centered care by offering open communication and empathy may mitigate the implications of social isolation on social relationships.

Information regarding patients’ preferences for the mode of information delivery was limited. However, one study reported a strong patient preference for face-to-face education and information brochures, compared to using video-assisted learning modes [34]. Conversely, many patients may have a preference to receive information through an informational video or Internet-based material [40]. Therefore, identifying and tailoring the mode of delivery of information to patients is of great importance as it may improve their comprehension, understanding, and perceptions about their MDRO infection, as well as their engagement and decision-making processes.

### Limitations

Several limitations and weaknesses that may arise in this review must be acknowledged. Firstly, the findings derived from the systematic review majorly depend on self-reported data which may be subject to response bias. Additionally, the studies were conducted in various countries, where contact isolation measures may differ and lead to difficulty in generalizing the findings.

## 5. Conclusions

MDRO infections are associated with various degrees of physical and psychological impact on patients and physicians. The physical impacts have been well documented; however, the psychological impact on patients is commonly overlooked. Psychological distress may be demonstrated in several ways, encompassing, but not limited to, anxiety, depression, loneliness, stigma, and anger. The physical toll that MDRO infections have on patients, especially when compounded with several adverse psychological effects, pose a threat to optimum patient care. By implementing a holistic approach tailored to patient needs, such as such as providing comprehensive patient education on the effects of MDROs and offering mental health counseling to deal with symptoms of depression and anxiety brought on by isolation, some of the patients’ psychological distresses may be alleviated. Additional studies assessing patients’ experiences and perceptions are needed to universally improve patient care for patients with MDRO infections in contact isolation.

## Figures and Tables

**Table 1:** Keywords used to define the search strategy aided by the PICOS (population, intervention/exposure, context, outcome, and study design) framework.

PICOS Category	Keywords
Population	Young adult [MeSH]; middle age; age 80 and over; age 65 and older; hospitalized; inpatient [MeSH]
Intervention/exposure	MDRO, drug resistance; microbial [MeSH], multi drug resistant bacteria; multi drug resistant pathogen
Comparator	-
Outcome	Patient experience; patient attitude; effect on patients; patient well-being; patient psychology; patient mental being; emotional effect; patient perception; psychological aspects of illness
Study Design	Case-control study; observational study

**Table 2:** Characteristics and main findings of the included studies.

Author (Year of Publication)	Methodology	Population Characteristics	Control Characteristics	Main Findings
Barratt et al. (2011) [28]	Qualitative design, semi-structured interviews.	10 adult patients hospitalized with MRSA infection in single rooms for at least 3 days.	N/A	Stigma, contamination, anger, frustration, and guilt were experienced by patients during MRSA isolation. Patients pursued greater insight into infection prevention and MRSA, recognizing the significance of PPE for others’ safety.
Catalano et al. (2003) [19]	Matched cohort testing. HAM-A and HAM-D were used to assess anxiety and depression levels.	27 patients aged 18 or above, infected with MRSA or VRE.	20 patients with an infectious disease not requiring isolation.	Depression and anxiety scores were both higher in the isolated, infected group compared to controls (*p* < 0.01) after 1 and 2 weeks.
Day et al. (2011) [29]	Retrospective cohort study.	4782 patients on contact precaution due to MDRO infection.	N/A	Contact precautions were associated with depression (odds ratio (OR) 1.4, 95% confidence interval (CI) 1.2–1.5) but not with anxiety (OR 0.8, 95% CI 0.7–1.1) in the non-ICU population.
Day et al. (2013) [20]	Prospective frequency-matched cohort study.	238 patients infected with *Clostridioides difficile* or other MDRO were placed in contact precautions.	290 patients with *Clostridioides difficile* or other MDRO not placed in contact precaution.	Three days following contact precautions, isolated patients showed no increase in depression levels. Contact precautions were also unrelated to happiness, sadness, worry, or anger as reflected by a VAMS score of 10%.
Evans et al. (2003) [21]	Matched cohort observation, questionnaire using Likert scale, retrospective review incidence.	485 isolated patients, of which 58% were infected with MRSA, VRE, *Clostridioides difficile*, multidrug-resistant *Acinetobacter* spp., or multidrug-resistant *S. maltophilia*.	1002 non-isolated patients in ICU setting.	Patients in isolation were seen less by healthcare professionals compared to non-isolated patients (*p* < 0.01).
Findik et al. (2012) [22]	Non-randomized quasi-experimental study. Data acquired (HADS-A (anxiety) and HADS-D (depression)) and patient information form.	60 patients with an MDRO * infection were isolated in a single room for 5 days.	57 non-isolated patients with a hospital infection diagnosis.	No statistically significant difference in anxiety and depression was found between isolated and non-isolated patients. However, females and those with lower income levels in the isolated group had higher levels of depression.
Gaube et al. (2023) [23]	Matched case-control study.	118 patients with contact isolation due to VRE, MRSA, or multidrug-resistant gram-negative bacteria infections	149 matched controls who were non-isolated (matched by hospital ward, sex, condition severity, age, and length of stay).	Patients under contact isolation reported greater dissatisfaction than non-isolated patients, particularly due to a lack of information provision regarding their MDRO diagnosis.
Goldsack et al. (2014) [30]	Mixed methods retrospective evaluation of patients diagnosed with MRSA infection.	211 patients in contact isolation due to MRSA infection, with 32 patients being selected for the questionnaire. Mean age: 61.	N/A	41% of patients reported contact isolation impacted their hospital stay, citing concerns about transmission, fewer visitors, feelings of anger, and contamination. Additionally, 28% experienced distress from isolation, feeling stigmatized, neglected, and having limited rights and privileges.
Kennedy and Hamilton (1997) [24]	Cross-sectional matched control study	16 patients infected with MRSA, aged 18–65.	MRSA-negative matched controls (matched for age, sex, level of injury, and time since admission or injury).	50% of the participants felt contact isolation worsened their mood, while 30% found it helpful in coping with their illness. Despite overall psychological well-being remaining mostly unchanged, isolated patients reported a significant rise in anger.
Lindberg et al. (2009) [31]	Qualitative design.	14 interviews of patients infected with MRSA.	N/A	Patients encountered fears and daily limitations due to MRSA colonization and felt a responsibility to prevent infection spread. Patient experiences with healthcare workers varied.
Newton et al. (2001) [32]	Qualitative, semi-structured interviews.	19 patients infected with MRSA hospitalized and placed in source isolation.	N/A	Patients had limited knowledge of MRSA’s impact, however, half deemed it “serious”. Most were uncertain about its origin and the purpose of isolation, despite seeing its pros and cons. Isolation did not appear to affect them psychologically.
Skyman et al. (2010) [33]	Qualitative, semi-structured interviews.	6 adult patients hospitalized in single rooms due to MRSA infection. All patients have been isolated for at least one week.	N/A	Contact isolation caused participants to feel confined, stigmatized, ashamed, and isolated. Patients criticized staff for not washing their hands regularly. Patients complained about being misinformed and confused regarding their MRSA diagnosis.
Smith and Ray-Barruel (2022) [34]	Bedside interviews with structured questionnaires.	30 patients with MRSA, VRE, or ESBL with a median age of 67 years	N/A	70% of patients were unaware of their infection and 23.3% were unsure of its duration. There was a clear preference for face-to-face education (96.7%) and brochures (86.7%) over phone calls (33.3%) and videos (0%). Communication and knowledge gaps exacerbated detrimental psychological effects, including fear and loneliness.
Soon et al. (2013) [25]	Cross-sectional matched control. HADS, MSPSS, PSS, and Euroqol Questionnaire tests were used.	20 patients in isolation due to MDRO * infection were compared to 20 non-isolated controls.	20 infected patients not in contact isolation.	A statistically significant increase in depression (t = 3.731, *p* < 0.01) and anxiety (t = 4.841, *p* < 0.001) was reported in patients in contact isolation compared with the controls.
Stelfox et al. (2003) [26]	Two matched cohorts	78 patients isolated due to MRSA infection.	156 non-isolated patients with other infectious disorders.	Patients in contact isolation were more likely to have their vital signs incompletely recorded or have days with no vital sign recordings at all (*p* = 0.08).
Tarzi et al. (2001) [27]	Cross-sectional matched control. The Abbreviated Mental Score, The Barthel Index, The Geriatric Depression Scale, and The Profile of Mood State tests were used.	22 MRSA-infected patients. Patients older than 65 in isolation.	20 non-isolated, MRSA-negative patients.	Independent sample *t*-tests showed higher levels of anxiety (t = 2.98, *p* < 0.01) and depression (t = 3, *p* < 0.01) in the isolated group compared to the non-isolated group. No difference in anger levels was reported (t = 0.06, not significant).
Watson et al. (2023) [13]	A sequential exploratory mixed-methods study, with semi-structured, face-to-face interviews.	76 patients approached, of which 20 patients conducted the interviews. The majority were males, older than 52 years. Patients had documented CPE or VRE infection.	N/A	Just 35% of staff reported that they would know exactly what to do if a patient was CPE-positive. Patients were confused by staff practices and inconsistent isolation protocols. Several individuals were not adequately aware of their MDRO diagnosis due to insufficient information. Written materials or verbal discussions with healthcare professionals were suggested as better information sources.

* Specific MDRO infections were not reported. HAM-A: Hamilton Anxiety Rating Scale; HAM-D: Hamilton Depression Rating Scale; HADS: Hospital Anxiety and Depression Scale; MSPSS: Multidimensional Scale of Perceived Social Support; PSS: Perceived Stress Scale; MDRO: Multidrug-Resistant Organism; MRSA: Methicillin-Resistant *Staphylococcus aureus*; VRE: Vancomycin-Resistant Enterococci; ESBL: Extended Spectrum Beta Lactamase; CPE: Carbapenemase-Producing Enterobacteriaceae.

## Data Availability

The original contributions presented in this study are included in the article and Supplementary Materials. Further inquiries can be directed to the corresponding author.

## Supplementary Materials

The following supporting information can be downloaded at: https://www.mdpi.com/article/10.3390/pathogens13090817/s1, Table S1: The Newcastle–Ottawa scale for quality assessment of cohort studies; Table S2: The Newcastle–Ottawa scale for quality assessment of case-control studies; Table S3: JBI critical appraisal checklist for cross-sectional studies; Table S4: JBI critical appraisal checklist for qualitative studies; Table S5: JBI critical appraisal checklist for quantitative studies; Table S6: The Mixed Methods Appraisal Tool version 2018 appraisal checklist for mixed-methods studies.
