# Supplementary material for: Patient Experiences and Perceptions with Infections Due to Multidrug-Resistant Organisms: A Systematic Review
Source: Pathogens. 2024 Sep 22;13(9):817. doi: 10.3390/pathogens13090817 (PMC11435282; doi:10.3390/pathogens13090817)
Supplement: Supplementary file 1 [file pathogens-13-00817-s001.zip › pathogens-3213344-supplementary.pdf]

Table S1: The Newcastle-Ottawa scale for quality assessment for cohort studies

Author (Year)	Selection				Comparability	Outcome			Final Score*
	Representativeess of the exposed cohort	Selection of the non-exposed cohort	Ascertainment of exposure	Demonstration that outcome of interest was not present at start of study		Assessment of outcome	Was follow-up long enough for outcomes to occur	Adequacy of follow up of cohorts	
Catalano et al., (2003)	*	*	*	*	**	-	*	*	8/9
Evans et al., (2003)	*	*	*	*	-	-	*	*	7/9
Stelfox et al., (2003)	*	*	*	*	**	*	*	*	9/9
Day et al., (2013)	*	*	*	*	**	*	-	*	8/9

*Scores equal or greater than 7 were considered to show that studies were of high quality

Table S2: The Newcastle-Ottawa scale for quality assessment for case-control studies

Author (Year)	Selection				Comparability	Exposure			Final Score*
	Is the case definition adequate?	Representativeness of the cases	Selection of the controls	Definition of controls		Assessment of exposure	Same method of ascertainment for cases and controls	Non-response rate	
Smith & Ray-Barruel (2022)	*	*	*	*	**	-	*	*	8/9

*Scores equal or greater than 7 were considered to show that studies were of high quality

Table S3: JBI critical appraisal checklist for cross-sectional studies

Checklist Item	Tarzi et al., (2001)	Kennedy & Hamilton (1997)	Soon et al., (2013)
Were the criteria for inclusion in the sample clearly defined?	Yes	Yes	Yes
Were the study subjects and the setting described in detail?	Yes	Yes	Yes
Was the exposure measured in a valid and reliable way?	Yes	Yes	Yes

Were objective, standard criteria used for measurement of the condition?	Yes	Yes	Yes
Were confounding factors identified?	No	Yes	Yes
Were strategies to deal with confounding factors stated?	Yes	Yes	Yes
Were the outcomes measured in a valid and reliable way?	Yes	Yes	Yes
Was appropriate statistical analysis used?	Yes	Yes	Yes

Table S4: JBI critical appraisal checklist for qualitative studies

Checklist Item	Newton et al., (2001)	Barratt et al., (2010)	Skyman et al., (2010)	Smith & Ray-Barruel (2022)	Lindberg et al., (2009)
Is there congruity between the stated philosophical perspective and the research methodology?	Yes	Yes	Yes	Yes	Yes
Is there congruity between the research methodology and the research question or objectives?	Yes	Yes	Yes	Yes	Yes
Is there congruity between the research methodology and the methods used to collect data?	Yes	Yes	Yes	Yes	Yes
Is there congruity between the research methodology and the representation and analysis of data?	Yes	Yes	Yes	Yes	Yes
Is there congruity between the research methodology and the interpretation of results?	Yes	Yes	Yes	Yes	Yes
Is there a statement locating the researcher culturally or theoretically?	No	No	Unclear	No	No
Is the influence of the researcher on the research, and vice-versa, addressed?	Unclear	No	Yes	Yes	Unclear
Are participants, and their voices, adequately represented?	Yes	Yes	Yes	Yes	Yes
Is the research ethical according to current criteria or, for recent studies, and is there evidence of ethical approval by an appropriate body?	Yes	Yes	Yes	Yes	Yes
Do the conclusions drawn in the research report flow from the analysis, or interpretation, of the data?	Yes	Yes	Yes	Yes	Yes

Table S5: JBI critical appraisal checklist for quantitative studies

Checklist Item	Fidnik et al., (2012)
Is it clear in the study what is the "cause" and what is the "effect"	Yes
Was there a control group?	Yes
Were participants included in any comparisons similar?	Yes
Were the participants included in any comparisons receiving similar treatment/care, other than the exposure or intervention of interest?	Not applicable
Were there multiple measurements of the outcome, both pre and post the intervention/exposure?	Yes
Were the outcomes of participants included in any comparisons measured in the same way?	Yes

Were outcomes measured in a reliable way?	Yes
Was follow-up complete and if not, were differences between groups in terms of their follow-up adequately described and analyzed?	Not applicable
Was appropriate statistical analysis used?	Yes

Table S6: The Mixed Methods Appraisal Tool version 2018 appraisal checklist for mixed method studies

Methodological quality criteria	Goldsack et al., (2014)	Watson et al., (2023)
Is there an adequate rationale for using a mixed methods design to address the research question?	Yes	Yes
Are the different components of the study effectively integrated to answer the research question?	Yes	Yes
Are the outputs of the integration of qualitative and quantitative components adequately interpreted?	Yes	Yes
Are divergences and inconsistencies between quantitative and qualitative results adequately addressed?	Yes	Yes
Do the different components of the study adhere to the quality criteria of each tradition of the methods involved?	Yes	Yes
